# Accidents in the Extraction of Teeth

**Published:** 1846-09

**Authors:** 


*Accidents in the Extraction of Teeth.*—Although accidents in the extraction of teeth are now of much less frequent occurrence than formerly, when skilful dentists were more rare, we do, nevertheless, occasionally hear of the infliction of serious injuries in the performance of this operation. Dr. Oliver Holmes, of this city, presented us, a few days since, with the three left superior molars and the entire alveolar border, including the floor and a portion of the outer and inner walls of the antrum, which had been fractured in an attempt to extract the first molar, by an individual unacquainted with the manner of performing this operation. Having fixed a key instrument to the tooth, as he supposed, which he wished to remove, he applied to it a sudden and tremendous force, when the whole alveolar ridge gave way. Discovering the injury which he had inflicted, he disengaged the instrument and dismissed his patient, with the uncomfortable assurance that he did not think it safe to extract the tooth, and who, when he left him was not aware of the real nature and extent of the injury he had sustained. Perceiving soon after, that the three back teeth were much loosened, he applied to Dr. Holmes, who, upon examination, at once discovered that the first, second and third molars, together with the entire alveolar ridge were completely detached, and only retained in the mouth by the soft parts with which they were connected. On separating them from these they were removed with a very slight effort.

We could mention, if it were necessary, a number of like accidents which have come to our knowledge, that have occurred in attempts to extract teeth, by persons uninstructed in the proper manner of performing the operation, and with facts such as these before us, it is astonishing that any one should apply to an individual for the removal of a tooth, who was not known to be skilled in this branch of practice. A few years since a girl about twelve or thirteen years of age was sent to us for our advice in relation to an injury she had received from an effort which had been made to extract her second right inferior molar, by a distinguished physician of this city, to whom she had applied. The jaw had been fractured, and from neglect, the parts had ulcerated, and the fractured extremity of the posterior portion, at the time we saw her, was protruding and in a necrosed condition.

